# Examining the Psychometrics of the National HIV Behavioral Surveillance measure for community HIV-related Stigma

**DOI:** 10.1007/s10461-021-03378-7

**Published:** 2021-07-20

**Authors:** Angel B Algarin, Gladys E Ibañez, David W Forrest, Monica Faraldo, Emma C Spencer, Lorene Maddox

**Affiliations:** 1. Division of Infectious Diseases and Global Public Health, University of California San Diego, 9500 Gillman Drive La Jolla, CA, 92093, USA; 2. Department of Epidemiology, Florida International University, 11200 SW 8th St. AHC5-478, Miami, FL 33199, USA; 3. Department of Anthropology, University of Miami, PO Box: 248106, Coral Gables, FL 33124, USA; 4. Department of Anthropology, University of Miami, PO Box: 248106, Coral Gables, FL 33124, USA; 5. Florida Department of Health, 4052 Bald Cypress Way, Tallahassee, FL 32399, USA; 6. Florida Department of Health, 4052 Bald Cypress Way, Tallahassee, FL 32399, USA

**Keywords:** Psychometrics, HIV-related stigma, men who have sex with men, Florida

## Abstract

The research tested the psychometrics of the Centers for Disease Control and Prevention’s National HIV Behavioral Surveillance(NHBS) community HIV-related stigma scale. Data was from men who have sex with men(MSM) NHBS cycles conducted 2011-2017 in Miami-Dade, Florida among N=1,455 participants. MSM were cis-gender male, 18+ years old, reported lifetime oral/anal sex with a male, and lived in Miami-Dade County. We assessed reliability using Cronbach’s alpha and McDonald’s omega, determined factors using principal factor analysis, and assessed construct validity using five a priori hypotheses. The scale was unidimensional, had questionable internal reliability (α=0.68, ω=0.69), and met four of five a priori hypotheses in the expected direction. Correlations were medium-weak in strength and only one was consistently met. Future iterations of the NHBS survey should consider replacing the 4-item community HIV-related stigma scale with an instrument that has superior internal reliability, measures multiple HIV-related stigma dimensions, and demonstrates stronger evidence of validity.

## RESUMEN

La investigación evaluó la psicometrías de la escala comunitaria de estigma relacionada con el VIH de La Vigilancia del Comportamiento Nacional del VIH de los Centros de Control y la Prevención de Enfermedades (National HIV Behavioral Surveillance, NHBS por sus siglas en Ingles). Los datos fueron de hombres que tienen sexo con hombres (HSH) ciclos NHBS realizados 2011-2027 en Miami-Dade, Florida entre N=1455 participantes. Los HSH eran hombres cisgénero, mayores de 18 años, reportando haber tenido sexo oral/anal de toda la vida con un hombre y vivían en el condado de Miami-Dale. Evaluamos la confiabilidad usando el alfa de Cronbach y el omega de McDonald, determinamos los factores usando el análisis de factores principales y evaluamos la validez de constructo usando cinco hipótesis a priori. La escala era unidimensional, tenía una fiabilidad interna cuestionable (α=0.68, ω=0.69), y cumplía cuatro de cinco hipótesis a priori en la dirección esperada. Las correlaciones fueron de intensidad media-dϩbil y solo una se cumplió de manera consistente. Las iteraciones futuras de la encuesta NHBS debería considerar reemplazar la escala comunitaria de estigma relacionada con el VIH de 4 ítems por un instrumento que tenga una confiabilidad interna superior, mida múltiples dimensiones del estigma relacionado con el VIH y demuestre una evidencia mas solida de validez.

## INTRODUCTION

Men who have sex with men (MSM) continue to face a disproportionate burden of new HIV diagnoses in the United States (U.S.), where in 2018 MSM comprised 66% of all new HIV diagnoses.^(1)^ Although between 2014-2018, MSM experienced an overall 7% decrease in HIV incidence, there were large racial disparities present within this rate change; where white MSM experienced a 16% decrease, but rates among Black and Hispanic MSM remained stable.^(1)^ In light of the disproportionate burden of HIV among MSM, it is of increased importance to study factors related to HIV outcomes in this population.

Beginning in 2003, the Centers for Disease Control and Prevention (CDC) created the National HIV Behavioral Surveillance (NHBS) survey to conduct behavioral surveillance among persons with increased risk of HIV acquisition.^(2)^ The survey measures behavioral risk factors (e.g. sexual behaviors, substance use), HIV testing and prevention behaviors, and other community perception variables like HIV- and gay-related stigma. The NHBS provides an important tool for systematic monitoring of the impact of the National HIV/AIDS strategy as well as providing a behavioral context for trends in HIV incidence.^(2)^ Despite the NHBS’s importance, little has been done to assess the reliability and validity of key measurements within the survey, namely HIV- and gay-related stigma.

HIV-related stigma is significantly deleterious to HIV-related health outcomes.^(3–6)^ As described by Turan et al. (2017), HIV-related stigma is comprised of 4 sub-dimensions including enacted, internalized, anticipated, and community stigma.^(7)^ Enacted stigma can be described as discrimination based on one’s HIV status, while anticipated stigma is the fear of the consequences after revealing one’s HIV status.^(7)^ Internalized stigma are negative feelings about oneself due to one’s HIV status, while community stigma is the perceived negative feelings of people living with HIV (PLWH) within one’s community.^(7)^

Of the 4 sub-constructs comprising HIV-related stigma, the NHBS survey only measures community HIV-related stigma. Previous research using NHBS data has found significant association between community HIV-related stigma and decreased age,^(8)^ non-white race/ethnicity,^(8,9)^ HIV status miss-reporting,^(10)^ antiretroviral therapy (ART) non-adherence,^(11)^ and decreased preexposure prophylaxis (PrEP) awareness/willingness.^(12)^ However, the measurement of community HIV-related stigma was inconsistent between studies, where some studies used single items, converted the ordinal item into a binary variable, and/or utilized a mean item score. Consistency in measurement operationalization is important as it allows for better comparability of results between NHBS sites. Despite the deleterious effect of community HIV-related stigma, no research has examined the validity of the scale used in the survey. Establishing validity and reliability of a scale is important as it ensures researchers are measuring what they intend to measure and that they measure the same construct each time.

The current research was conducted to test the psychometrics of the NHBS community HIV-related stigma scale. We hypothesized the scale would be unidimensional given findings from previous studies on HIV stigma^(13,14)^ where community HIV-related stigma items loaded onto their own factor. We anticipated that the scale would have good internal consistency that would be temporally consistent. Finally, we hypothesized that the community HIV-related stigma scale would have weak to moderate positive correlation with distress and a positive HIV test result, and would have a weak to moderate negative correlation with community gay-related tolerance, PrEP awareness, and ART adherence.

## METHODS

The NHBS survey is an anonymous serial, cross-sectional survey currently conducted in 22 cities across the U.S. to assess the association of behavioral factors on HIV surveillance data. NHBS is conducted among three different key groups: men who have sex with men, people who inject drugs, and heterosexuals at increased risk for HIV. We used data from the CDC NHBS survey conducted in Miami-Dade County, Florida among MSM in 2011, 2014, and 2017 (MSM3, MSM4, MSM5, respectively). MSM were recruited using venue-based time-space sampling (VBS), described in previous studies.^(15)^

To be eligible for participation, MSM had to identify as cis-gender male, 18+ years of age, report oral or anal sex with a male in their lifetime, able to complete the interview in English or Spanish, and report living in the Miami-Dade County, Florida. Study staff systematically approached and screened men to establish eligibility for participation. Eligible MSM consented and completed an interviewer-driven survey using a tablet computer. Questions assessed demographics, sexual and drug use behaviors, HIV prevention and testing behaviors, and perceived community stigma. All participants were offered an HIV test. Participants during MSM3 and MSM4 received $25 for completing the survey and $25 for completing the HIV test and $35 for completing the survey and $40 for completing the HIV test for MSM5.

### Community HIV-related Stigma

Community HIV-related stigma was measured using a scale adapted from two previous studies.^(13,14)^ The scale was comprised of 4 items with 5-point Likert style responses from “strongly disagree=1” to “strongly agree=5” (e.g. Most people in *Miami* think that people who got HIV through sex or drug use have gotten what they deserve.). The item, “Most people in *Miami* would support the rights of a person with HIV to live and work wherever they wanted to,” was reverse coded to be in the same direction as the other items. Total possible scores could range from 4-20, where higher scores indicated greater perceived community HIV-related stigma.

### Measurement of Construct Validity

#### Community gay-related tolerance

Community gay-related tolerance was measured with the item, “Most people in Miami are tolerant of gays and bisexuals,” using 5-point Likert options from “strongly disagree=1” and “strongly agree=5.”

#### Distress

Psychological distress was measured using the Kessler Psychological Distress scale (K6) (α=0.86)^(16)^ only in the MSM5 survey. The K6 scale contains 6 items with 5-point Likert style responses from “none of the time=0” to “all of the time=4” (e.g. During the past 30 days, how often did you feel hopeless?). Scores could range from 0-24 where higher scores indicated higher levels of distress.

#### HIV test result

All participants of MSM3-MSM5 were offered a rapid HIV test to be conducted at the end of the questionnaire. Participants were classified as testing positive (1) or negative (0). Those with an indeterminate test result were removed from the analyses.

#### PrEP Awareness

In the MSM3 survey, self-reported HIV-negative participants were asked the yes/no question, “Before today, have you ever heard of people who do not have HIV taking antiretroviral medicines, to keep from getting HIV?” In the MSM4 survey, self-reported HIV negative participants were asked the yes/no question, “Before today, have you ever heard of people who do not have HIV taking PrEP, the antiretroviral medicine taken every day for months or years to reduce the risk of getting HIV?” In the MSM5 survey, self-reported HIV-negative participants were asked the yes/no question, “Preexposure prophylaxis, or PrEP, is an antiretroviral medicine, such as Truvada, taken for months or years by a person who is HIV-negative to reduce the risk of getting HIV. Before today, have you ever heard of PrEP?”

#### ART Use

In MSM3-MSM5 surveys, self-reported HIV positive participants were asked the yes/no question, “Are you currently taking antiretroviral medicines to treat your HIV infection?”

### Demographics

Participants reported age (18–24, 25–34, 35–44, 45+), race (white, Black/African American, other), ethnicity (Hispanic, Non-Hispanic), U.S. (not including Puerto Rico) nativity (yes/no), sexual identity (heterosexual, homosexual, bisexual), and HIV-status (positive, negative). Socioeconomic status (SES) was stratified, based on previous literature into 3 levels (low, middle, high).^(11,17,18)^ Low SES was classified as earning <$25,000 per year or no high school diploma, medium SES was earning an income $25,000-49,999 or high school diploma, high SES was earning an income of >$50,000 and a college degree.

### Analyses

All analyses were conducted in SAS Studio®. Frequencies and percentages were calculated to display pooled and stratified participant characteristics of MSM3–MSM5 cycles. After, principal factor analysis was conducted with *promax* rotation to determine if the community HIV-related stigma scale had any latent factors. An oblique rotation was used as we hypothesized that if more than one factor arose, they would be correlated. Scree test and eigenvalues of >1 and factor loading of >0.30 were used to determine factors and loadings respectively.^(19)^ Internal reliability of the scale was assessed using Cronbach’s alpha and McDonald’s omega using the developed SAS macros by Hayes & Coutt (2020).^(20)^ We include Cronbach’s alpha to allow for comparison of our results to other studies, given its widespread use throughout the literature. However, use of Cronbach’s alpha to assess reliability has some limitations outlined in previous literature,^(20–22)^ particularly in scales with less than 10 items,^(22)^ leading us to also include the preferred reliability measure of McDonald’s omega. Pearson’s and Spearman’s correlation were conducted to determine correlations established in the a priori hypotheses, depending on whether the variable was continuous or ordinal, to determine construct validity using the *proc corr* statement in SAS. We handled missing data using listwise deletion. α was set to 0.05.

## RESULTS

After removing participants with incomplete community HIV-related stigma measures (n=167), MSM3—MSM5 studies successfully recruited a pooled N=1,455 participants (MSM3 n=503, MSM4 n=544, MSM5 n=408, respectively). The majority of the pooled sample was 25–34 years of age (34.0%), Hispanic (67.6%), and tested HIV-negative (75.5%). More on sample characteristics can be found in Table I.

The Cronbach’s alpha for the pooled sample of the 4-item community HIV-related stigma measure was α=0.68, ranging from α=0.64-0.71 between cycles. The McDonald’s omega for the pooled sample of the 4-item community HIV-related stigma measure was ω=0.69, ranging from ω=0.65-0.72 between cycles. None of the items were found to be deleterious to the internal reliability. Factor analysis indicated scale unidimensionality (pooled sample: Eigenvalue=1.29, Difference 1.36) and all items had factor loadings above 0.40 (Table II).

Overall, the pooled sample reported low-moderate levels of community HIV-related stigma (Table II) with a total scale average of 11.51±2.99 and a range of 4–20. In the pooled sample, two of the four a priori hypotheses were met where community HIV-related stigma was negatively correlated with community gay-related tolerance (r_s_=−0.22; p<0.001) and PrEP awareness (r_s_=−0.07; p=0.024). In MSM3 and MSM4, one of four a priori hypotheses were met where community HIV-related stigma was negatively correlated with community gay-related tolerance (r_s_=−0.15; p<0.001, r_s_=−0.22; p<0.001, respectively). In MSM5, three of five a priori hypotheses were met, where community HIV-related stigma was positively correlated with distress (r=0.23; p<0.001) and negatively correlated with community gay-related tolerance (r_s_=−0.29; p<0.001) and testing HIV positive (r_s_=−0.09; p=0.044). All met hypotheses were in the appropriate direction, with the exception of testing HIV positive as negatively associated with community HIV-related stigma. Based on Cohen’s guidance for interpreting correlation coefficients,^(23)^ the strength of the significant relationships was between small and medium (Table III).

## DISCUSSION

This was the first study to test the psychometrics of the community HIV-related stigma scale used in the NHBS survey among MSM. The unidimensional scale had acceptable internal consistency, but only consistently reached one of four a priori hypotheses in establishing construct validity (not including distress from MSM5). Though at different points the scale was significantly correlated with constructs directly associated with HIV-related stigma (e.g. distress, community gay-related tolerance, HIV test result, and PrEP awareness) the correlations were weaker than expected, and it was not significantly correlated with manifestations of community HIV-related stigma, such as current ART use. The findings of this study may imply the need to use a new HIV-related stigma scale in the NHBS survey. The national NHBS sample may be large enough to find significant differences between community HIV-related stigma and HIV-related health outcomes; however, the use of the community HIV-related stigma scale to find local level differences, where sample sizes are smaller, may limit the ability to use tailored data to support Florida’s plan to eliminate HIV transmission and reduce HIV-related deaths^(24)^ and inform state leaders on potential targets for stigma reduction interventions among MSM. Moreover, as previous research suggests,^(6,25,26)^ other dimensions of HIV-related stigma not measured in the NHBS survey (e.g. enacted, internalized, and anticipated) may have a larger effect size with HIV-related health outcomes.

Additionally, the reliability of the community HIV-related stigma scale was questionable, suggesting that other measures with superior internal reliability should be considered. One measure to consider is the Multiple Discrimination Scale (MDS) developed by Bogart et al. (2013) that measures multiple dimensions of stigma related to HIV, race/ethnicity, and sexuality all with good internal reliability (α>0.83).^(27)^ Another measure to consider is the 12-item HIV stigma scale,^(28)^ adapted from the 40-item Berger HIV stigma scale,^(29)^ that measures internal, enacted, community, and anticipated stigma with good internal reliability (α>0.80). Based on the results, future iterations of the NHBS survey should consider replacing the 4-item community related stigma scale with an instrument that 1) has superior internal reliability, 2) measures multiple dimensions of HIV-related stigma, 3) shows stronger evidence of validity.

Overall, perceived community HIV-related stigma was low-moderate in the sample. The majority of the items had mean scores that fell between “disagree” and “neither agree nor disagree”; however, the item, “Most people in *Miami* would discriminate against someone with HIV,” had a score that had a mean that fell between “neither agree nor disagree” and “agree.” Future qualitative research should further examine whom participants considered “most people in Miami” as well as what specific types of discrimination would be enacted (i.e. name calling, gossip, violence, etc.).

### Limitations

This study had a number of limitations. First, the sample was comprised only of MSM in Miami-Dade County, Florida, meaning that the findings may not be generalizable to MSM in other NHBS study sites. Future research utilizing the nationwide NHBS sample may provide stronger evidence of instrument psychometrics. Secondly, participants recruited by the VBS recruitment method may not be generalizable to MSM who do not attend the targeted venues. Thirdly, we were unable to examine correlations between the community HIV-related stigma scale and other HIV stigma measures, as the main study was not designed to validate the scale. Lastly, participants who were willing to participate in a study surrounding HIV behavior may have had lower perceptions of community HIV-related stigma.

Despite the limitations, our study had some key strengths. Firstly, the sample used to validate the community HIV-related stigma scale was diverse, with the majority of participants falling into racial/ethnic HIV risk categories. Additionally, examining reliability and validity by NHBS cycle allowed for examination of instrument stability through time.

## CONCLUSIONS

We successfully tested the psychometrics of the NHBS survey community HIV-related stigma scale. Questionable internal reliability and poor correlation with HIV-related health outcomes suggest the potential necessity for a new scale to measure HIV-related stigma. If deemed necessary, the future scale should be an instrument that 1) has superior internal reliability, 2) measures multiple dimensions of HIV-related stigma, 3) shows stronger evidence of validity.

## Figures and Tables

**Table I.** NHBS sample demographics of MSM recruited in Miami-Dade County, Florida 2011—2017 (N=1,455)

	Total	Cycle 3	Cycle 4	Cycle 5
				
Age Category	N=1,455	n=503	n=544	n=408
18-24 years of age	318 (21.9)	153 (30.4)	101 (18.6)	64 (15.7)
25-34 years of age	495 (34.0)	150 (29.8)	220 (40.4)	125 (30.6)
35-44 years of age	301 (20.7)	100 (19.9)	112 (20.6)	89 (21.8)
45+ years of age	341 (23.4)	100 (19.9)	111 (20.4)	130 (31.9)
Race				
White	874 (65.8)	286 (67.1)	289 (58.4)	299 (73.5)
Black	377 (28.4)	114 (26.8)	186 (37.6)	77 (18.9)
Other/Multi-Racial	77 (5.8)	26 (6.1)	20 (4.0)	31 (7.6)
Ethnicity				
Hispanic	984 (67.6)	339 (67.4)	324 (59.6)	321 (78.7)
Non-Hispanic	471 (32.4)	164 (32.6)	220 (40.4)	87 (21.3)
Nativity				
U.S. Born (not including Puerto Rico)	696 (47.8)	268 (53.3)	302 (55.5)	126 (30.9)
Non-U.S. Born	759 (52.2)	235 (46.7)	242 (44.5)	282 (69.1)
Sexual Identity				
Heterosexual	19 (1.3)	5 (1.0)	7 (1.3)	7 (1.7)
Homosexual	1146 (78.8)	403 (80.3)	422 (77.6)	321 (78.7)
Bisexual	289 (19.9)	94 (18.7)	115 (21.1)	80 (19.6)
Self-report HIV Status				
Negative	1089 (80.8)	365 (81.7)	419 (81.2)	305 (79.2)
Positive	259 (19.2)	82 (18.3)	97 (18.8)	80 (20.8)
HIV test result				
Negative	1089 (75.5)	385 (77.0)	396 (73.7)	308 (76.1)
Positive	346 (24.0)	111 (22.2)	139 (25.9)	96 (23.7)
Indeterminate	7 (0.5)	4 (0.8)	2 (0.4)	1 (0.2)
Socioeconomic Status				
Low	652 (44.9)	208 (41.5)	273 (50.2)	171 (41.9)
Medium	492 (33.9)	190 (37.9)	162 (29.8)	140 (34.3)
High	309 (21.3)	103 (20.6)	109 (20.0)	97 (23.8)
Community Gay-related Tolerance				
Strongly Agree	205 (14.1)	55 (11.0)	85 (15.6)	65 (15.9)
Agree	760 (52.3)	241 (48.1)	292 (53.7)	227 (55.6)
Neither Agree, not Disagree	264 (18.2)	109 (21.8)	95 (17.5)	60 (14.7)
Disagree	198 (13.6)	82 (16.4)	65 (12.0)	51 (12.5)
Strongly Disagree	26 (1.8)	14 (2.8)	7 (1.3)	5 (1.2)
Distress Score (mean±std)	---	---	---	5.3±5.0
PrEP Aware (self-reported, HIV-negative only)	492 (41.2)	85 (20.2)	179 (40.1)	228 (69.5)
Current ART use (self-reported, HIV-positive only)	223 (90.3)	70 (88.6)	80 (87.9)	73 (94.8)

**Table II.** Factor analysis results and reliability estimates of National HIV Behavioral Surveillance Community HIV-related Stigma Scale Miami-Dade County, Florida 2011—2017 (N=1,455)

	Total (N=1,455)				MSM 3 (n=503)				MSM 4 (n=544)				MSM 5 (n=408)			
Likert items, 5-point range from “strongly disagree=1” to “strongly agree=5”	Mean ± std	Item-Total Corr.	Factor Loading	α if deleted	Mean ± std	Item-Total Corr.	Factor Loading	α if deleted	Mean ± std	Item-Total Corr.	Factor Loading	α if deleted	Mean ± std	Item-Total Corr.	Factor Loading	α if deleted
Most people in *Miami* would discriminate against someone with HIV. Do you…	3.54 ± 1.03	0.46	0.56	0.62	3.56 ± 1.06	0.49	0.59	0.63	3.60 ± 1.03	0.42	0.53	0.58	3.42 ± 1.00	0.47	0.55	0.65
Most people in *Miami* would support the rights of a person with HIV to live and work wherever they wanted to. Do you…*	2.61 ± 0.98	0.39	0.47	0.66	2.72 ± 1.02	0.41	0.50	0.67	2.56 ± 0.97	0.35	0.44	0.63	2.54 ± 0.93	0.40	0.48	0.69
Most people in *Miami* would not be friends with someone with HIV. Do you…	2.63 ± 1.03	0.54	0.65	0.57	2.70 ± 1.02	0.56	0.67	0.58	2.66 ± 1.05	0.51	0.63	0.52	2.50±1.01	0.55	0.66	0.60
Most people in *Miami* think that people who got HIV through sex or drug use have gotten what they deserve. Do you…	2.73 ± 1.13	0.47	0.58	0.61	2.80 ± 1.11	0.46	0.55	0.65	2.83 ± 1.12	0.42	0.52	0.58	2.53 ± 1.14	0.55	0.66	0.61
Total	11.51 ± 2.99	---	---	α=0.68	11.80 ± 3.05	---	---	α=0.70	11.65 ± 2.90	---	---	α=0.64	10.99 ± 2.98	---	---	α=0.71
				ω=0.69				ω=0.70				ω=0.65				ω=0.72

* Item reverse coded

**Table III.** 4-item Community HIV-related stigma correlation with external constructs

	Total	MSM 3	MSM 4	MSM 5
	Correlation; p	Correlation; p	Correlation; p	Correlation; p
Community gay-related tolerance^a^	−0.22; <0.001	−0.15; <0.001	−0.22; <0.001	−0.29; <0.001
Distress^b^	---	---	---	0.23; <0.001
HIV test result^a,c^	−0.03; 0.237	0.00; 0.929	−0.00; 0.997	−0.09; 0.044
PrEP awareness^a^	−0.07; 0.024	−0.00; 0.969	−0.03; 0.539	−0.00; 0.941
ART use^a^	0.03; 0.686	0.01; 0.939	0.02; 0.865	0.06; 0.607

a. Spearman Correlation

b. Pearson Correlation

c. Those with indeterminate test results were removed from analyses

## Data Availability

N/A
